# Genetic variability of Hepatitis B virus in Morocco

**Published:** 2011-01-10

**Authors:** B Kitab, R Afifi, A Essaid El Feydi, M Benazzouz, H Salih Alj, K Rebbani, I Brahim, O Derdab, Y Cherradi, M Hassar, S Ezzikouri, S Benjelloun

**Affiliations:** 1 Laboratoire des Hépatites Virales, Institut Pasteur du Maroc, Casablanca, Morocco; 2 Service de Médecine C, CHU Ibn Sina, Rabat, Morocco; 3 Laboratoire de Recherche sur les Lipoprotéines et l’athérosclérose, Faculté des Sciences, Université Hassan II, Mohammedia, Casablanca, Morocco

## Background

Morocco is a medium-level epidemic country for Hepatitis B Virus (HBV). However, little is known about clinical, virologic and phylogenetic features of HBV infection. The aim of the present study is to determine the HBV genetic variability, and its association with clinical outcome and severity of disease in Moroccan HBV carriers.

## Methods

The study included 250 chronic HBV infection patients at different stages of liver disease (156 male and 94 female). Serum samples were tested for serological markers and HBV DNA levels. The HBV Surface and core promoter/precore regions were amplified and directly sequenced. The clinical, virologic and phylogenetic characteristics were investigated.

## Results

The mean age of patients was 44 ± 12.2. Most of them were HBeAg negative (90%). The mean HBV DNA was 475104 ± 160591 UI/mL. Phylogenetic analysis identified 90% isolates in genotype D and 10% in genotype A. Most genotypes D isolates belonged to subgenotype D7 (80%) followed by subgenotype D1 (25%) and one isolate belonged to subgenotype D2. All genotype A strains belonged to subgenotype A2 and specified subtype *adw2*. In genotypes D strains, subtypes *ayw2* (91.7%), *ayw3* (3.3%) and *ayw4* (3.3%) were identified. A significance prevalence of mutations in the Major Hydrophilic Region (MHR) of HBsAg was found with P120T/S the most frequent. In the core promoter region, the most frequent mutations are G1757A (48.9%), T1773C (42.8%), C1766GÂ /T (40.8%), T1753V (30.7%) and A1762T/G1764A (24.4%). In the precore region, the most common mutations are G1896A (55.1%) and G1899A (34.6%). Double mutation in the core promoter A1762T/G1764A was found more frequently in HCC patients than that in non HCC patients (66.6 % *vs* 16.2%; p<0.001). In addition, the prevalence of C1653T, T1753V, and G1862T mutations was significantly higher in HCC patients compared with non HCC. However, the prevalence of the G1896A precore mutation was not different between patients with HCC and HBV carriers without HCC (55.5% *vs* 55%; p>0.05).

## Conclusion

We described for the first time that Subgenotype D7/ayw2 is the most predominant in Morocco. The high frequency of mutations in the core promoter in patients with HCC indicates their association with severity of infection.

